# Hyperthyroidism After Allogeneic Hematopoietic Stem Cell Transplantation: A Report of Four Cases

**DOI:** 10.4274/jcrpe.2295

**Published:** 2015-12-03

**Authors:** Erdal Sağ, Nazlı Gönç, Ayfer Alikaşifoğlu, Barış Kuşkonmaz, Duygu Uçkan, Alev Özön, Nurgün Kandemir

**Affiliations:** 1 Hacettepe University Faculty of Medicine, Department of Pediatrics, Ankara, Turkey; 2 Hacettepe University Faculty of Medicine, Department of Pediatric Endocrinology, Ankara, Turkey; 3 Hacettepe University Faculty of Medicine, Department of Pediatric Hematology, Ankara, Turkey

**Keywords:** Hyperthyroidism, autoimmune, Hematopoietic stem cell transplantation, Bone marrow transplantation

## Abstract

Hematopoietic stem cell transplantation (HSCT) is the only curative treatment for many hematological disorders, primary immunodeficiencies, and metabolic disorders. Thyroid dysfunction is one of the frequently seen complications of HSCT. However, hyperthyroidism due to Graves’ disease, autoimmune thyroiditis, and thyrotoxicosis are rare. Herein, we report a series of 4 patients who were euthyroid before HSCT but developed hyperthyroidism (3 of them developed autoimmune thyroid disease) after transplantation.

**WHAT IS ALREADY KNOWN ON THIS TOPIC?**

Thyroid dysfunction is frequently seen after hematopoietic stem cell transplantation, however, hyperthyroidism is one of the rarest complications.

**WHAT THIS STUDY ADDS?**

Hyperthyroidism developing after hematopoietic stem cell transplantation may vary in etiology, severity and need of treatment.

## INTRODUCTION

Hematopoietic stem cell transplantation (HSCT) remains the only curative treatment for many inherited and acquired pediatric hematological disorders such as haemoglobinopathies, bone marrow failure disorders, primary immunodeficiencies, and metabolic disorders.

Thyroid dysfunction is one of the frequently seen complications of HSCT. Several kinds of thyroid disease such as hypothyroidism, euthyroid sick syndrome, autoimmune thyroiditis, Graves’ disease, and thyroid tumors have been reported so far. Hypothyroidism, which is seen in nearly 40% of patients, is the most common thyroid disease, and it is especially more prevalent among patients receiving total body irradiation (1,2,3,4,5). Graves’ disease, autoimmune thyroiditis, and thyrotoxicosis are seen rarely, and the underlying mechanisms are either transfer of donor auto-reactive immune cells or immune dysregulation and immune reconstitution secondary to graft-versus-host disease (GVHD) (6). Herein, we report a series of 4 patients who were euthyroid before HSCT but developed hyperthyroidism (3 of them developed autoimmune thyroid disease) after transplantation.

## CASE REPORT

### Case 1

A ten-month-old female was diagnosed with beta-thalassemia major and underwent bone marrow transplantation from her HLA-matched mother when she was 29 months of age. Neutrophil and platelet engraftments were observed on the 15th and 33rd days of transplantation, respectively (Table 1).

On post-transplant day 25, she had acute GVHD, presenting with nodular and maculopapular rash, and methylprednisolone was initiated. She did not respond to steroid and cyclosporine A (CsA) treatment. Mycophenolate mofetil was added to the treatment regimen. At +5th month, she had a seizure with magnetic resonance imaging findings compatible with posterior reversible encephalopathy syndrome (PRES). CsA treatment was replaced by tacrolimus. At +19th month, a mosaic pattern at thorax high-resolution computed tomography (HRCT) was detected, a bronchoscopy was performed, and she was diagnosed as bronchiolitis obliterans. Owing to the failure of steroid, mycophenolate, and tacrolimus therapy, a skin biopsy was performed and chronic GVHD was diagnosed. Twenty cycles of extracorporeal photopheresis (ECP) was initiated.

On post-transplant month 40, when the patient was 5 years old, increased sweating was noticed; her heart rate was 125/min and blood pressure was 100/65 mmHg. There was no palpitation, exophthalmos, tremor, or other symptoms of hyperthyroidism. Her thyroid function tests (Table 2) revealed hyperthyroidism with a free triiodothyronine (fT3) level of 8.6 pmol/L (3.8-6.0 pmol/L). Levels for the following were: free thyroxine (fT4) 17.02 pmol/L (7.86-14.41 pmol/L), thyroid-stimulating hormone (TSH) 0.04 µIU/mL (0.34-5.6 µIU/mL), thyroglobulin 97.8 ng/mL (1.15-50 ng/mL), anti-thyroid peroxidase antibody (anti-TPO) 36.3 IU/mL (0-9 IU/mL), anti-TSH receptor antibody 34.4 IU/L (<1 IU/L), and anti-thyroglobulin antibody <0.9 IU/mL (0-4 IU/mL). Spot urine iodide level was 3.8 µg/dL (10-20 µg/dL). Her thyroid ultrasonography (USG) was normal. There was no family history of any thyroid or autoimmune disease. However, her mother (donor) was diagnosed to have Hashimoto’s thyroiditis when tested following her daughter’s diagnosis. The patient was treated with propranolol and methimazole.

### Case 2

A fifteen-year-old female presented with high-grade fever, diarrhea, recurrent infections, hepatosplenomegaly, and pancytopenia. Her brother had died with a diagnosis of secondary hemophagocytic syndrome due to Epstein-Barr virus infection. Six out of 8 diagnostic criteria for hemaphagocytic syndrome were present in the patient, and the diagnosis was established (7). She was found to be in an accelerated phase of the disease, and the HLH-2004 protocol was initiated. Bone marrow transplantation was performed from the patient’s HLA-matched 16-year-old sister. Neutrophil and platelet engraftments were observed on the 11th and 15th days, respectively (Table 1).

On post-transplant day 9, the patient developed a maculopapular rash, which was assessed as indicative of acute GVHD. Methylprednisolone was initiated. She responded well and the steroid treatment was tapered and stopped.

Over time, the patient was observed to develop hypertension, neutropenic fever, and grade 3-4 mucositis as HSCT complications.

On the post-transplant month 5, tachycardia was observed (heart rate: 120/min), but she had no hypertension (blood pressure: 110/70 mmHg), palpitation, exophthalmos, tremor, or other symptoms of hyperthyroidism. Her thyroid function tests (Table 2) revealed hyperthyroidism with a fT3 level of 11.58 pmol/L (3.8-6.0 pmol/L), fT4 28.98 pmol/L (12-22 pmol/L), TSH 0.02 µIU/mL (0.34-5.6 µIU/mL), anti-TPO antibody 7 IU/mL (0-9 IU/mL), anti-TSH receptor antibody <1 IU/L (<1 IU/L), and anti-thyroglobulin antibody <0.9 IU/mL (0-4 IU/mL). Her thyroid USG was normal. She was followed without treatment and had subclinical hypothyroidism on post-transplant 8th month (TSH: 7.92 µIU/mL, fT3: 4.66 pmol/L, fT4: 12.86 pmol/L). Later, on the follow-up at month 22, she had another hyperthyroidism attack with a fT3 7.13 pmol/L (3.8-6.0 pmol/L), fT4 23.23 pmol/L (12-22 pmol/L), TSH 0.07 µIU/mL (0.34-5.6 µIU/mL), and anti-TPO antibody 54.5 IU/mL (0-9 IU/mL). She again was followed without treatment for 4 months, at which time her thyroid hormone levels returned to normal. She is now euthyroid at post-transplant month 60 with a fT3 level of 4.59 pmol/L (3.8-6.0 pmol/L), fT4 10.94 pmol/L (7.86-14.41 pmol/L), and TSH 2.57 µIU/mL (0.34-5.6 µIU/mL).

### Case 3

This male patient had presented with recurrent infections, moniliasis, anemia, and thrombocytopenia to another medical center at age one month and had been hospitalized with a diagnosis of severe combined immunodeficiency disorder (SCID). HSCT was done from his HLA-full matched sister. T cell engraftment was observed after HSCT; however, thrombocytopenia continued. He was then re-evaluated and diagnosed as Wiskott-Aldrich syndrome. When he was 7 years old, a second bone marrow transplantation was done from his sister in our institution. Neutrophil and platelet engraftments were observed on the 13th and 18th days, respectively. He did not have any major HSCT complication (Table 1).

On post-transplant month 7, he had mild tachycardia (heart rate: 125/min), but he had no hypertension (TA: 90/60 mmHg), palpitation, exophthalmos, tremor, or other symptoms of hyperthyroidism. His thyroid function tests (Table 2) revealed hyperthyroidism with a fT3 level of 11.3 pmol/L (3.8-6.0 pmol/L), fT4 28.17 pmol/L (12-22 pmol/L), TSH 0.02 µIU/mL (0.34-5.6 µIU/mL), anti-TPO antibody 38.0 IU/mL (0-9 IU/mL), anti-thyroglobulin antibody <0.9 IU/mL (0-4 IU/mL), and anti-TSH receptor antibody <1 IU/L (<1 IU/L). His thyroid USG revealed thyroiditis without any nodules. He was treated only with propranolol. He was euthyroid 5 months later (post-transplant month 12) with a fT3 6.25 pmol/L (3.8-6.0 pmol/L), fT4 14.59 pmol/L (7.86-14.41 pmol/L), and TSH 2.41 µIU/mL (0.34-5.6 µIU/mL) without any anti-thyroid treatment.

### Case 4

A sixteen-year-old male presented with left upper extremity pain, anemia, and thrombocytopenia. A bone marrow aspiration was done and he was hospitalized with a diagnosis of acute myeloid leukemia (AML). HSCT was performed at remission from his HLA-full matched sister. Neutrophil and platelet engraftments were observed on the 14th and 17th days, respectively. On post-transplant month 16, there was a mosaic pattern at thorax HRCT. Later on, he developed diarrhea. Steroid and CsA treatment was initiated with a diagnosis of chronic GVHD (Table 1).

On the post-transplant month 17, weight loss was noted. He had no tachycardia (heart rate: 98/min), hypertension (blood pressure: 100/60 mmHg), palpitation, tremor, exophthalmos, or other symptoms of hyperthyroidism. His thyroid function tests (Table 2) revealed hyperthyroidism with a fT3 6.27 pmol/L (3.8-6.0 pmol/L), fT4 18.19 pmol/L (7.86-14.41 pmol/L), TSH 0.08 µIU/mL (0.34-5.6 µIU/mL), thyroglobulin 140.6 ng/mL (1.15-50 ngmL), anti-TPO antibody 94 IU/mL (0-9 IU/mL), anti-thyroglobulin antibody <0.9 IU/mL (0-4 IU/mL), and anti-TSH receptor antibody 5 IU/L (<1 IU/L). His spot urine iodide level was 16.5 µg/dL (10-20 µg/dL). His thyroid USG revealed mild thyroiditis without any nodules. He was followed without treatment. Three weeks later, he was found to have severe weight loss (10 kg in three weeks). Physical examination revealed tachycardia and fine tremors in both hands. His thyroid gland was palpable without any signs of nodules. His thyroid function tests were repeated with a fT3 6.71 pmol/L (3.8-6.0 pmol/L), fT4 46.6 pmol/L (7.86-14.41 pmol/L), and TSH 0.04 µIU/mL (0.34-5.6 µIU/mL). A diagnosis of Hashimoto’s thyroiditis was made, and methimazole treatment was initiated. Subsequently, the methimazole dose was tapered gradually and treatment was ceased after 6 months when he became euthyroid.

## DISCUSSION

In our institution, the total number of HSCT in pediatric cases was 313 at the time of this study. Herein, we report four cases of hyperthyroidism after allogeneic HSCT.

Thyroid dysfunction is an important problem in patients treated with HSCT, and several kinds of thyroid disorder such as hypothyroidism, euthyroid sick syndrome, autoimmune thyroiditis, Graves’ disease, and even thyroid tumors have been reported. Borgström and Bolme (1) reported a series of 35 allogeneic bone marrow transplant patients; nearly 89% of them had signs of disturbance in the thyroid axis, mostly due to total body irradiation. In a series of 95 autologous stem-cell transplant recipients from Italy, 15 patients (16%) had transient subclinical hyperthyroidism and 29 patients had transient low T3 syndrome. In another report from the same center evaluating 40 patients, 47.5% were found to have thyroid dysfunction diagnosed as low T3 syndrome, chronic thyroiditis, transient subclinical hyperthyroidism, and subclinical hypothyroidism (4,5). In a different series of 57 stem cell recipients from Japan, 24 had euthyroid sick syndrome (15 of these patients became euthyroid within 8.4 months) and 8 patients had thyrotoxicosis. All of the thyrotoxicosis cases were transient with a mean duration of 2 months; 7 patients with thyrotoxicosis developed hypothyroidism during the follow-up period, with a mean of 12 months after HSCT (2). Au et al (3) reported that 4 out of 222 HSCT patients had autoimmune thyroid disease after treatment and all of these cases had a specific haplotype, HLA-A2-B46-DR9.

In animal models, transfer of HSC can induce remission of some autoimmune conditions. However, HSC can also cause autoimmunity when given to previously healthy mice in experimental studies (6,8). There are previous reports of patients with autoimmune diseases such as systemic lupus erythematosus, Crohn’s disease, autoimmune thrombocytopenia, and others who have had complete remission after HSCT which had been done for their associated malignancies (9). On the other hand, there are also cases developing autoimmune diseases such as psoriasis, vitiligo, myasthenia gravis, and autoimmune thyroiditis after receiving HSCT (8).

Hyperthyroidism after allogeneic bone marrow transplantation is rare and is presumably mediated by the transfer of immunocompetent donor lymphocytes to the recipient by HSCT (10). It can occur either by the transfer of pathogenic auto-reactive lymphocyte clone from donor to recipient, or by GVHD (6). There are reports of such cases in the relevant literature (11,12,13,14).

In our patients, we can easily rule out the possibility of radiation injury to the thyroid gland because none of our patients had total body irradiation. In our first case, the donor was an anti-TPO antibody positive, anti TSH receptor antibody negative euthyroid mother. However, the patient had hyperthyroidism with anti-TPO antibody and anti TSH receptor antibody positive, which suggest that the hyperthyroidism did not develop simply because of the transfer of adoptive immunity. The donor was her mother, she was screened after the patient had hyperthyroidism and was found to have a euthyroid Hashimoto’s disease. With all these findings and genetic susceptibility, immune dysregulation as a result of GVHD or other factors may have led to expansion of auto-reactive lymphocytes to those which were silent in the donor. Mulligan et al (15) reported a patient with autoimmune hyperthyroidism associated with chronic GVHD after HSCT, but there was no other organ involvement. They speculated that organ-specific GVHD, specific only against thyroid gland, was responsible for the autoimmune hyperthyroidism (15). This same mechanism may have been at work in our second patient, since she was euthyroid before HSCT, had hyperthyroidism without any autoantibody detected after treatment, and her donor was also autoantibody-free but had acute GVHD. Case 3 had a similar clinical and autoantibody status, however, he did not experience either acute or chronic GVHD. Case 4 had hyperthyroidism due to Hashimoto’s thyroiditis only 1 month after he was diagnosed to have chronic GVHD, his and his donor’s anti-TPO antibody and anti-thyroglobulin antibody were positive.

We conclude that both immune dysregulation and adoptive immunity transfer may have played a role in the development of autoimmune thyroid disease in our patients, but the exact mechanisms need to be elucidated. HSCT can cause thyroid dysfunction more frequently than expected, therefore, the thyroid status of each HSCT patient should be screened before and after the treatment. Further studies are warranted to assess the requirement of screening for thyroid autoantibodies before or after HSCT.

## Figures and Tables

**Table 1:**
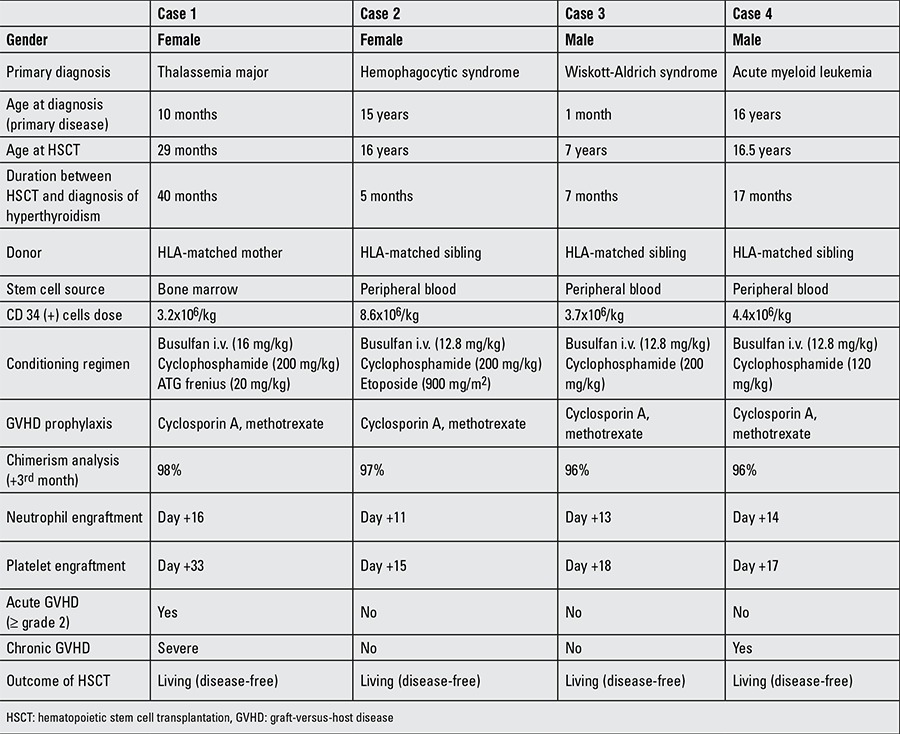
Clinical features of the patients

**Table 2:**
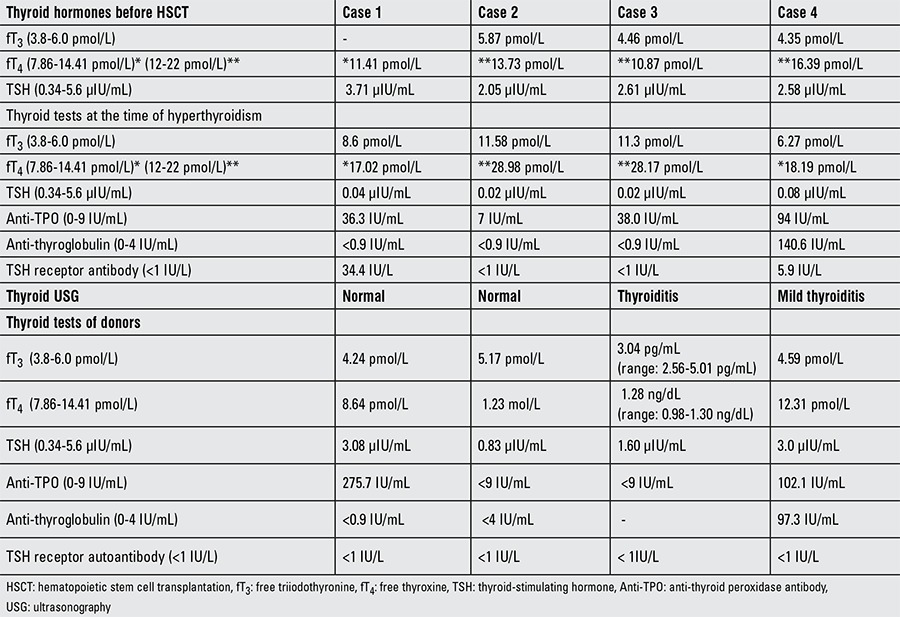
Laboratory tests for hyperthyroidism in the cases and their donors
